# Can a text message a week improve breastfeeding?

**DOI:** 10.1186/s12884-014-0374-2

**Published:** 2014-11-06

**Authors:** Danielle Gallegos, Rebekah Russell-Bennett, Josephine Previte, Joy Parkinson

**Affiliations:** School of Exercise and Nutrition Sciences, Queensland University of Technology, Kelvin Grove, Brisbane, 4059 Australia; QUT Business School, Queensland University of Technology, George St, Brisbane, 4001 Australia; UQ Business School, University of Queensland, St Lucia, Brisbane, 4072 Australia; Griffith Business School, Griffith University, Nathan, Brisbane, 4111 Australia

**Keywords:** Breastfeeding, Mobile phone, Text messaging, Intervention studies, Coping behaviour

## Abstract

**Background:**

Breastfeeding is recognised as the optimal method for feeding infants with health gains made by reducing infectious diseases in infancy; and chronic diseases, including obesity, in childhood, adolescence and adulthood. Despite this, exclusivity and duration in developed countries remains resistant to improvement. The objectives of this research were to test if an automated mobile phone text messaging intervention, delivering one text message a week, could increase “any” breastfeeding rates and improve breastfeeding self-efficacy and coping.

**Methods:**

Women were eligible to participate if they were: over eighteen years; had an infant less than three months old; were currently breastfeeding; no diagnosed mental illness; and used a mobile phone. Women in the intervention group received *MumBubConnect,* a text messaging service with automated responses delivered once a week for 8 weeks. Women in the comparison group received their usual care and were sampled two years after the intervention group. Data collection included online surveys at two time points, week zero and week nine, to measure breastfeeding exclusivity and duration, coping, emotions, accountability and self-efficacy. A range of statistical analyses were used to test for differences between groups. Hierarchical regression was used to investigate change in breastfeeding outcome, between groups, adjusting for co-variates.

**Results:**

The intervention group had 120 participants at commencement and 114 at completion, the comparison group had 114 participants at commencement and 86 at completion. *MumBubConnect* had a positive impact on the primary outcome of breastfeeding behaviors with women receiving the intervention more likely to continue exclusive breastfeeding; with a 6% decrease in exclusive breastfeeding in the intervention group, compared to a 14% decrease in the comparison group (p < 0.001). This remained significant after controlling for infant age, mother’s income, education and delivery type (p = 0.04). Women in the intervention group demonstrated active coping and were less likely to display emotions-focussed coping (p < .001). There was no discernible statistical effect on self-efficacy or accountability.

**Conclusions:**

A fully automated text messaging services appears to improve exclusive breastfeeding duration. The service provides a well-accepted, personalised support service that empowers women to actively resolve breastfeeding issues.

**Trial registration:**

Australian New Zealand Clinical Trials Registry: ACTRN12614001091695.

## Background

Breastfeeding is universally acknowledged as the optimal method for feeding infants with well-established short and long term benefits [1-5]. Current international best-practice recommendations for breastfeeding are for infants to be exclusively breastfed until six months of age, with recognition that any breastfeeding for as long as possible affords benefits [5]. In Australia, the recommendations are for exclusive breastfeeding to six months and continuation to 12 months of age and beyond [6]. Despite recognition of breastfeeding as optimal, rates in developed countries remain low. In Australia breastfeeding initiation is relatively high at 96% but rapidly declines, with only 69% and 21% of Australian infants being predominantly breastfed at three and five months respectively; and only 39% and 15% exclusively breastfed at the same age (breastfeeding practices as defined by the World Health Organization) [7,8]. These figures are comparable for the United States [9] but higher than in the UK where exclusive breastfeeding at 6 months is around 1% [10].

Decisions on infant feeding are complex. The majority of women know that breastfeeding is the best option for their infants [11], however in order to continue breastfeeding women need to manage a range of physical and psychological factors that will impact on breastfeeding duration [12]. In addition to non-modifiable factors (age, education and income of mother), there are a range of sociocultural, environmental and personal determinants that influence breastfeeding duration and exclusivity; including breastfeeding intention, self-efficacy and social support [13,14]. Self-efficacy or the confidence to perform a particular task to achieve a desired outcome is known to be a strong predictor of breastfeeding duration [15]. Coping is defined as psychological and behavioural efforts to minimise stress associated with a particular condition. Coping can give an indication of how an individual seeks or uses social support or feels supported [16]. Breastfeeding is considered a learned skill and can be stressful to establish and maintain, however, to date, coping strategies have not been investigated in relation to breastfeeding. Active coping (problem-focussed, seeks social support) is considered to be more effective than emotions focused coping and this is the first known application of the concept to breastfeeding [16,17].

The provision of postnatal support by both professionals and peers has been identified as a key element in improving breastfeeding duration and exclusivity [18]. The findings of a recent systematic review concluded that: women benefited more from ongoing support in areas with high initiation rates; support could be provided by a combination of peer and professionals; face-to-face consultations were more likely to succeed; and interventions where women are expected to initiate contact are unlikely to be effective [19]. A review of telephone interventions indicated that proactive telephone support in the early postnatal period could potentially impact on breastfeeding duration and exclusivity [20]. Other research has concluded that there is encouraging evidence that the use of digital technologies, primarily web-based interventions, will improve breastfeeding behaviours [21,22]. There have been no known studies on the impact of using mobile phone technologies to support breastfeeding practice by improving self-efficacy and coping. Mobile health technologies are being effectively used for a range of other health conditions: smoking cessation [23-25]; weight maintenance [26]; physical activity promotion [25,27-29]; diabetes and other long-term illness management [30,31]; and maternal health [32]. There has been a significant penetration of mobile phone technologies worldwide with access to mobile networks now estimated to be available to 96% of the world’s population [33]. This makes mobile phones attractive modes of delivery for health interventions. This paper reports on *MumBubConnect*, an innovative branded, two-way text messaging service.

The first objective of this research was to evaluate the ability of an automated mobile phone text messaging intervention to increase any breastfeeding rates. The second objective was to test if a mobile support intervention improved breastfeeding self-efficacy and positive coping strategies.

## Methods

### Intervention content

*MumBubConnect* (MBC) sent women a single text message once a week for eight weeks, asking them how their breastfeeding was proceeding to all women in the intervention group. It then asked for a standard response to which women received an automated reply. Women received a magnet and wallet card with the responses required. A response indicating some level of distress (for example, keyword of ‘worried’, ‘confused’ or ‘down’) prompted a trained Australian Breastfeeding Association (ABA) breastfeeding counsellor to make an outbound call within 24 hours. The text message responses were about normalising common issues and problems (such as sore nipples, milk oversupply or under-supply), providing active solutions and affirming positive behaviour. The messages were developed through focus groups with women [34] and with input from the ABA, a public health nutritionist and marketing experts. A creative director assisted with the development of the branding. Table 1 provides an example of the key words and follow-up responses.

**Table 1:** **MumBubConnect text and response examples**

**Key word texted by mother**	**Automated response received**
	How’s breastfeeding going today? If you have stopped, text STOP to let us know.
Happy	Great! Breastfeeding is a skill, and it sounds as though you and your baby have learned fast! Call 1800 686 2 686 if needed.
Average	Breastfeeding takes time for you and baby to master and you are doing fine so far. Call 1800 686 2 686 if you need help.
Sore	Maybe vary feeding position, and make sure baby has a full mouthful of breast. Call 1800 686 2 686 for help.
Engorged	Let your baby feed more often, and make sure breasts are drained. Use a cold pack to help, and call 1800 686 2 686.
Always (Feeding)	When baby’s suck slows down and breast is drained, take a break, change activity. Call 1800 686 2 686 for help.

### Breastfeeding definitions

Throughout this paper the WHO definitions for breastfeeding are used [8]. In particular, exclusive breastfeeding, when infants receive breastmilk only (oral rehydrating solution, medicines, vitamins and minerals acceptable); and predominant breastfeeding when the infant receives breastmilk and certain other fluids (water, water-based drinks, fruit juice, ritual fluids, medications) [8] Partial breastfeeding refers to an infant receiving both breastmilk and artificial formula [2].

### Participants

A non-concurrent, prospective, comparison trial was conducted where we recruited 120 and 114 women into intervention and comparison groups respectively. Eligible women were recruited for the intervention during a three week period over September and October 2010, via national broadcast media (radio interviews and mainstream press releases). Initially, women, who self-selected into the intervention, were going to be randomised into control and intervention groups but due to the overwhelming response for support, a control group was deemed unethical, therefore all women who registered were allocated to the intervention group as a convenience sample. As a result, a second group was recruited to act as a comparison group between August and October 2012 via social media. Women were eligible if they were: over eighteen years of age; had an infant less than three months of age; were currently doing any breastfeeding ; did not have a diagnosed mental illness; and used a mobile phone (of any type). Completion of the online questionnaire on registration was taken as consent to participate.

**Figure 1:**
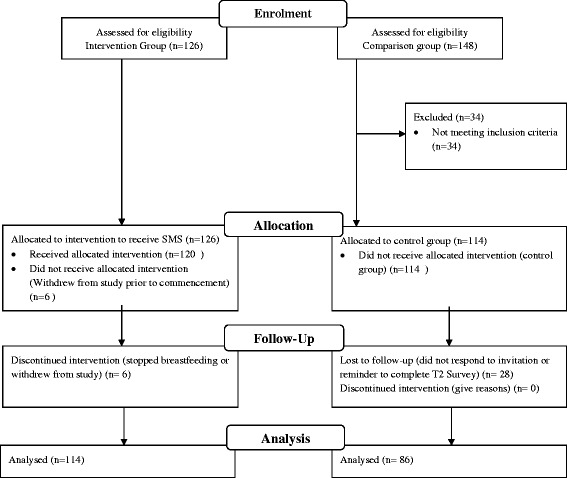
**CONSORT flow diagram.**

Women in the intervention group (n = 120) were directed to register at a website, complete an online questionnaire and then consent to receiving a text message once a week for eight weeks, see Figure 1 (CONSORT diagram). The trial commenced on October 18 2010 and finished on December 13 2010. They were also directed to a facebook page which provided forum for women to talk further about their involvement. At week nine women were asked to complete a post-intervention survey (n = 114) and received a small gift (an iTunes voucher to the value of $10). Women who did not respond to the post-intervention survey were followed up two weeks later. Women in the comparison group (n = 114) were emailed a link to the questionnaire at project commencement and again at week nine. Non-respondents at week nine were contacted two weeks later to increase response rates. On completion of the second questionnaire (n = 86) women received the same small gift.

### Data collection

The questionnaire included a range of measures drawn from the literature. These are outlined in Table 2. The questionnaire for the intervention group also included open-ended process evaluation questions at the end of the trial.

**Table 2:** **Measures used pre and post for intervention and comparison groups**

**Items**	**Comment**	**Cronbach’s alpha**
Demographic	Including postcode or usual residence, education level achieved, annual income, relationship status, other children, web/phone use. [7,35]	
Infant details	Including, birth day to calculate age in days, type of delivery (vaginal or caesarean), weight. [7,35]	
Mother details	Including breastfeeding intentions prior to birth, return to work, ethnicity. [7,35]	
Current infant feeding practices	24 hour recall, indicated as best practice. Breastfeeding definition in accordance with WHO indicators. [7,8,35]	
Self-efficacy	Previously validated breastfeeding self-efficacy measured by 13 items using a five point Likert scale anchored by ‘not at all confident’ to ‘always confident’. [36]	.92
Social support	As a function of self-efficacy. Also included current levels of support from family, peers, professionals and organisations measured using a seven point Likert scale with summated score anchored by ‘no support’ and ‘lots of support’. [36,37]	.97
Coping checklist	Identifies active and emotions-focussed coping, and self-accountability as a contributor to efficacy.	
	Ways of Coping Checklist (WCCL), five point Likert scale anchored by ‘rarely’ and ‘frequently’ identifies five main forms of coping:	
	• problem-focused (13 items);	.86
	• wishful thinking (6 items);	.86
	• seeks social support (3 items);	.90
	• blamed self (3 items);	.89
	• avoidance (9 items).	.88
	Problem-focused + social support = active coping.	
	Wishful thinking + blamed self + avoidance = emotions focussed coping. [17,38,39]	
Emotions	Positive (hope, challenge) and	.85
	negative (fear, guilt, regret) emotions used 5 items using a 5 point Likert scale anchored by ‘not at all’ and ‘very much’ [39].	.79
Breastfeeding knowledge and awareness	Eight validated questions on knowledge and awareness [36,37].	

In-group and between group changes were assessed between baseline and at the completion of the second questionnaire. Differences in the group characteristics; age, socioeconomic status (using postcode to assess against the Socioeconomic Index for Advantage (SEIFA), [40] remoteness (using the Accessibility and Remoteness Index for Australia [41]), marital status, and age of infant were assessed. Breastfeeding practice was described as exclusive (EBF) or predominant (PBF), and assessed as an outcome. Changes were measured in social support, accountability, self-efficacy, active coping, emotions-focussed coping, and each of the coping constructs (problem-focussed, wishful thinking, seeks social support, blamed self, avoidance). Process evaluation data was gathered on: text messages sent and received; number of outward calls made by the breastfeeding counsellor; and qualitative comments on the acceptability and logistics of the text messaging service.

Statistical analyses were conducted using SPSS 21. T-tests were used to test for differences between groups for independent variables and changes in independent variables. Chi-square tests were used to test for differences between categories such as income and education. ANCOVA was used to test if the independent variable was having an effect and allowed the influence of the covariates to be controlled for during analysis. Hierarchical regression was used to investigate change in breastfeeding outcome, between groups, adjusting for co-variates. When controlling for covariates, factors that were significant at the bivariate level were adjusted for in the model.

### Ethics approval

This research had Queensland University of Technology Human Research Ethics Approval (0800000506, 1000000568, 1100000234).

## Results

### Sample characteristics

For the intervention group, 120 women were recruited and 114 women completed the second questionnaire (response rate of 95%). For the comparison group 114 women were recruited and 86 completed the second questionnaire (response rate 75%). Table 3 illustrates the demographic and breastfeeding characteristics of the two groups. Women were from a variety of backgrounds but were predominantly Anglo-celtic Australian, well-educated, had relatively high socio-economic status and lived in metropolitan areas. There was however, representation from women who were experiencing disadvantage related to socio-economic status or isolation.

**Table 3:** **Sample characteristics**

**Characteristic**	**Intervention group**	**Comparison group**	**p value** *
	**(n = 114)**	**(n = 86)**	
Average age of mother (years)	31 ± 4	30 ± 5	
Average age of infant at start of trial (days)	61 ± 30	47 ± 24	
Mother’s education (%)			
Year 10 or less	7	6	
Year 12	10	11	ns
Post highschool qual.	22	21	
University	61	63	
Don’t want to say	1	0	
Income (%)			
Less than $25 000 (AUD)	5	1	
$25-50 000	12	8	0.03
$50-100 000	40	37	
Over $100 000	35	40	
Don’t know or don’t want to say	7	13	
Index of advantage (decile)			
High advantage =7-10	47	52	ns
Med advantage =4-6	36	23	
Low advantage =1-3	17	24	
Rural/remote score			
1 (major city, highly accessible)	61	58	ns
2 (inner regional, accessible)	17	16	
3 (outer regional, mod. accessible)	14	19	
4 (remote, remote)	4	7	
5 (very remote, very remote)	4	0	
Relationship status	95	98	ns
Married - Yes			
C-Section (including elective and emergency)	33	31	ns
Yes			
Member of the Australian Breastfeeding Association	45	25	
Yes			

*Chi-square test was used to assess the proportional differences for categorical variables, whereas the student t test was used to assess the mean differences for continuous variables.

### Results

#### Impact of MBC on breastfeeding rates

The first objective was to test the ability of an automated mobile phone text messaging intervention to increase any breastfeeding rates. Table 4 provides a summary of the changes in breastfeeding practices between intervention and comparison groups. EBF rates in the intervention group decreased by 6% whereas rates in the comparison group decreased by 14%. The difference in change between the two groups was statistically significant. Hierarchical regression was used to assess levels of change in EBF between groups, after controlling for the influence of infant age, income, education and delivery type. Infant age, income, education and delivery type were entered at Step 1, explaining 3% of the variance in change in EBF. After entry of “group” (ie intervention vs comparison) at Step 2, the total variance explained by the model as a whole was 5%, F (5,186) = 1.95, p = 0.05. In the final model, only two of the variables were statistically significant, with “group” recording a higher β value of 6.23 (3.03), p = 0.04 and infant age recording a β value 0.10 (.05), p = 0.05.

**Table 4:** **Breastfeeding practice in-group and between group**

**Breastfeeding practice**	**Intervention group**		**Comparison group**		**Differences in changes between groups over time**
	**%**		**%**		
	**Time 1**	**Time 2**	**Time 1**	**Time2**	
Exclusive Breastfeeding	75	69*	91	77***	*
Predominant Breastfeeding	11	8	4	3***	ns
Partial feeding	14	20*	5	19***	ns
Not Breastfeeding	0	3	0	1	ns

*Significant at the <0.05 level, ***Significant at the <0.001 level.

### Impact of MBC to improve self-efficacy and active coping

The second objective was to test if a mobile support intervention improved breastfeeding self-efficacy and coping. Table 5 provides details of the variation in each of the constructs used in the Ways of Coping Checklist (WCCL), unadjusted for potential covariates. Mothers in the intervention group were significantly more likely to become problem focussed (p = 0.001) and to seek social support (p = 0.003); and less likely to blame self (p = 0.03), resort to wishful thinking (p = 0.004) or undertake avoidance compared to the comparison group (p = 0.001).

**Table 5:** **Ways of coping checklist (WCCL) constructs and their changes over time and between groups**

**Coping variable name**	**Intervention (IG) T1 mean (SD)**	**InterventionT2 mean (SD)**	**Change T1 and T2 for IG**	**Comparison (CG) T1 mean (SD)**	**ComparisonT2 mean (SD)**	**Change T1 and T2 for CG**	**Confidence intervals (change between groups over time)**	P-value*
Problem focused	3.38 (0.89)	3.71 (0.76)	+0.33	3.61 (0.67)	3.44 (0.34)	-0.17	-0.37,0.08	0.001
Seeks social support	3.64 (1.06)	3.86 (0.89)	+0.22	3.90 (0.83)	3.58 (0.38)	-0.32	-0.77, 0.16	0.003
Blamed self	1.73 (0.92)	1.88 (1.06)	+0.15	1.76 (0.99)	2.59 (0.59)	+0.83	0.03, 0.64	0.03
Wishful thinking	1.81 (0.94)	2.10 (1.04)	+0.29	1.99 (1.01)	2.79 (0.56)	+0.80	0.14, 0.69	0.004
Avoidance	1.63 (0.69)	1.81 (0.80)	+0.18	1.73 (0.76)	2.67 (0.47)	+0.94	0.37, 0.82	0.001

*significance of the difference in change over time between intervention and comparison group.

Changes in self-efficacy or possible factors that might contribute to changes in self-efficacy are presented within and between the intervention and comparison groups in Table 6. In the intervention group there were increases in all the variables. When viewing the differences between the summated scores over the nine week period, and between the groups, the intervention group exhibited a significant increase in active coping and a significantly lower increase in negative coping, when compared to the comparison group. Increases in perceived social support for the intervention group were greater and was almost statistically significant. When these variables are controlled for by socioeconomic status (using SEIFA), education, delivery type (vaginal or caesarean) and being an ABA member, active coping and emotion-focussed coping variations remain significant.

**Table 6:** **Changes over time and between groups of scores for coping, social support, self-efficacy, emotions and accountability**

**Variable name**	**Intervention group (IG) time 1 (T1) mean (SD)**	**Intervention group time 2 (T2) mean (SD)**	**Change between T1 and T2 for IG**	**Comparison group (CG) T1 mean (SD)**	**Comparison group T2 mean (SD)**	**Change T1 and T2 for CG**	**CI (change between groups over time)**	P-value*
Active coping	3.51 (0.89)	3.78 (0.76)	+0.33	3.76(0.65)	3.51(0.31)	-0.25	-0.75, -0.23	0.01
Emotion- focussed coping	3.28 (0.74)	3.07 (0.85)	-0.23	3.17(0.79)	2.32(0.46)	-0.86	0.32,0.76	0.001
Perceived social support	3.64(1.05)	3.86(0.88)	+.24	3.91(0.86)	3.89(0.68)	-0.02	0.01,-0.28	<0.001
Self-efficacy	4.00 (0.74)	4.15 (0.72)	+0.15	4.22(0.66)	4.29(0.67)	+0.07	-0.24,0.06	0.25
Positive emotions	4.23 (0.58)	4.35 (0.61)	+0.12	4.64(0.67)	4.73(0.51)	+0.09	-0.07,0.22	0.31
Negative emotions	1.28 (0.65)	1.37 (0.78)	+0.09	1.28(0.43)	1.30(0.46)	+0.02	-0.01,0.28	0.07
Accountability	4.36 (0.67)	4.38 (0.69)	+0.02	4.31(0.77)	4.49(0.71)	+0.18	-0.07,0.37	0.17

*significance of the difference in change over time between intervention and comparison group.

### Process evaluation results

Frequency of text message responses increased from a low of 48% in week one to 80% and above from week two, peaking at 86% in week 6. Across the eight weeks the mean response rate was 6.34 (1.99). The maximum time period for non-response for the majority of women was a week, that is the majority of women responded to the text before receiving the next one.

Initially there were difficulties with women remembering to use the key words to respond with 20% of responses being incorrect in week one decreasing to 11% at the end of the trial. The most common responses regarding improving the service were around being able to free text (that is, not rely on the key word responses), and increasing the number of texts received during the week. Nearly three-quarters of women (73%) would have liked the option of sending a message when they needed to; for ongoing communication, 61% preferred a text message, while 35% indicated a telephone call.

The perception of support and encouragement continued in evaluation of the messages with 57% of women indicating they agreed or strongly agreed to the statement that “the messages encouraged me to continue breastfeeding”; and 68% of women agreeing and strongly agreeing to “I felt I had support when I received the messages”. There was a wealth of responses from women in the open-ended question asking how the service made them feel; Table 7 provides a thematic summary of these responses with exemplar quotes where relevant.

**Table 7:** **Qualitative responses of women participating in the intervention**

**Themes (number of responses)**	**Exemplar quotes**
Supported (33)	“As though there was support there and as the reply came back - I felt confident and ok about everything again. The reply being instant made me feel like there was someone there to listen and help with advice”
	“Good that somebody was interested in my breastfeeding, and not because I asked them to be interested (like family are interested because you make them interested)”
	“When I messaged that I had an issue, the message of support was just as important as the suggestion of what to do”
Encouraged (16)	“Felt encouraged. No one else has been telling me I’m doing a great job, so even though it was a computer it still gave me a little lift. Made me sad also though that no one in my life has told me I’m doing a good job. I feel supported by people in my life in that they aren’t saying anything negative, but I’m not getting anything positive either. I did when I was seeing my LC, but now that feeding is going well I don’t see her anymore”
Good (12)	“Good about myself and having useful information sent to me”
Confident (5)	“They gave me a confidence boost”
Connected (5)	“Part of a group”

An outbound telephone call was received by 12% of women. The call was from a trained breastfeeding counsellor after there were some indications of not coping or distress. Just over half (51%) of women responded to every text message, only 10% responded to two or less text messages. Nearly half (46%) of women indicated they contacted either one of the identified helplines for advice or assistance.

## Discussion

This was a pilot study to test proof of concept for an automated two-way text messaging service to support women to breastfeed. We aimed to test the ability of an automated mobile phone text messaging intervention, as a means to provide support for breastfeeding women, to increase any breastfeeding rates and to improve self-efficacy towards breastfeeding and active coping. *MumBubConnect* increased exclusive breastfeeding rates however did not impact on any or predominant breastfeeding rates. *MumBubConnect* has demonstrated that mobile support was well accepted as a means of support and while there was no significant difference in self-efficacy there was a significant difference in positive/active coping. MBC has significantly reduced attrition in women exclusively breastfeeding, when compared to the comparison group. This remained significant after controlling for factors normally associated with breastfeeding duration. While the intervention was trialled in a group of women who were potentially more likely to breastfeed the ubiquitous nature of mobile phone technology means that the likelihood of MBC reaching more difficult to reach women (less likely to breastfeed) is high. We anticipate that the results of a larger clinical trial would demonstrate the effectiveness of MBC to significantly correlate to improving exclusive breastfeeding rates at four and six months of age, but also any breastfeeding representing optimal breastfeeding practices.

The women recruited for the MBC intervention and the comparison groups were more likely to breastfeed compared to women nationally. Given the available data collected in 2004 and 2010 indicates little if no change in breastfeeding rates at less than six months, it is unlikely that breastfeeding rates changed between the collection of data from the intervention and comparison groups [7,42]. For infants in the sample aged up to four months, 96% and 98% of infants were receiving any breastmilk in the trial and comparison group respectively, compared to 68.7% based on nationally collected data [7]. Women in the comparison group had a greater prevalence of exclusive breastfeeding at time one potentially due to the younger average age of infants and the higher income of women. Despite this, while the women in the intervention group were more likely to breastfeed, the intervention would appear to have had an impact on extending the duration of exclusive breastfeeding.

The literature demonstrates that all forms of extra support have an effect on exclusive breastfeeding duration. Professional and lay support separately and together have effects on prolonging breastfeeding, with lay support more effective in extending exclusive breastfeeding [18]. Face-to-face support appears to have a larger treatment effect than telephone support, although pro-active telephone support does improve breastfeeding duration and exclusivity [19,20]. Support only offered to women who seek assistance is unlikely to be effective [19]. All forms of support reviewed, relied however, on time provided by peers or professionals and were relatively intensive in terms of cost and infrastructure. MBC has the potential to provide the same or greater effects, which could be universally provided and is low-cost. This potential warrants further investigation.

Self-efficacy has been well documented as being a key determinant of breastfeeding duration, and this was not affected by MBC [36]. All women recruited to both the intervention and the comparison groups had high levels of self-efficacy and these were maintained over the nine week period. However, other factors are known to influence self-efficacy including higher perceptions of social support and physiologic and emotional states such as pain, anxiety and stress [43,44]. Given the significant results of factors contributing to self-efficacy, MBC could have a more profound impact among women with lower identified self-efficacy levels in the early post-partum period. The impact could also be dose-related, that is if the women received more text messages for a longer period of time the effect may have been greater and changes in self-efficacy may have been seen. These elements remain to be investigated.

To our knowledge the “Ways of Coping Checklist” has not been applied to breastfeeding behaviour. Previously, the WCCL in its earlier and revised forms has been used to investigate coping behaviours related to chronic or life-threatening illnesses, being a carer, and in managing burn-out, stress and anxiety [38,45-49]. Breastfeeding is a learned skill that can be challenging to manage and can create high levels of stress and anxiety and how women cope with this is potentially important in determining best methods of support. The indications are that the intervention assisted women in enabling them to problem solve and to seek additional support. In addition, they were less likely to blame themselves, undertake avoidance or engage in wishful thinking. MBC appears to have significantly increased engagement in active coping strategies; with women in the intervention increasing and women in the comparison group decreasing active coping. In addition, emotions-focussed coping increased in both groups but the increase for the women in the comparison group was almost three times that of women receiving the intervention. These are all indications that MBC has had a positive impact on empowering women to manage their breastfeeding experience by increasing active coping strategies.

Mobile technologies are increasingly used by individuals to extend and manage relationship connections with selected “experts”, as well as to gather information and marketing offers, anytime, anywhere [34]. Mobile phones may be useful in delivering health-related services because they are: personal (targeted and individualised); portable (always on and always-on-us); connected (human-to-human, human-to-machine); and intelligent (increased capacity at the mobile level) [50]. There are indications that mobile technologies offer opportunities for technology-enhanced social connections, that promote positive health behaviours [51]. The MBC intervention has provided evidence that women preferred this modality over other forms of telephone, web-based or face-to-face support due to: the personalised messages (even though they knew they were automated); being asked about how they were feeling, rather than admitting defeat and asking for help; responding on their terms, rather than being dictated by a telephone call or an appointment time. In response from the women, future iterations of the service may incorporate the ability to free text and to initiate a text when required.

This research has demonstrated that a mobile phone intervention has the potential to impact coping strategies of mothers to improve breastfeeding exclusivity and duration. Using mobile phone technologies provides an opportunity to broadly and cheaply provide an affordable, proximal, personalised and customised means for influencing breastfeeding behaviours. There is also scope to further explore the WCCL, emotions and accountability as constructs that could contribute to the explanation of breastfeeding behaviours.

### Limitations

This study was a proof of concept, that is, a mobile phone intervention could positively impact on breastfeeding behaviors. There are a number of limitations the most notable is that the trial was not randomised and the control and intervention groups were not run concurrently. While a randomised control trial was originally planned the overwhelming need expressed by women in the community for additional support meant that the researchers were uncomfortable in not providing the intervention to all women during the trial. In addition, the marketing component of the intervention made it difficult to limit exposure of the control group if run simultaneously. While there were limitations in the non-concurrent design there was also one distinct advantage. The MBC intervention had a significant social marketing component and was accompanied by significant media, delaying the control group reduced the likelihood of recognition of key messages from the intervention. Careful consideration will be given to the design of the effectiveness trial given the identified difficulties for population based interventions [52].

In addition, both intervention and comparison groups were pro-breastfeeding; and the samples were diverse but still weighted towards women with higher socioeconomic status. Recruitment via the media could have resulted in a volunteer bias.

This trial did not include an “attention” text, in other words it is unclear if it was the messages contained within the text or the fact that they received a text that created the effect. Any ongoing trial will need to carefully consider an attention text which does not provide information or problem solving with respect to feeding or aspects related to feeding. Finally, there was a higher attrition of women from the comparison group who were lost to follow up. This could have impacted on the groups but also provides indication that the MBC intervention actively engaged women over the eight weeks.

## Conclusions

MBC as a low-cost intervention has the potential to increase the duration of any and exclusive breastfeeding. The trial of proof of concept has demonstrated increases in women’s perception of coping and support. The wide use of mobile phone technology throughout low, middle and high income countries means that the concept has the potential for universal application. Extension of the trial beyond eight weeks to include breastfeeding duration at four and six months of age with a broader sample of women will also be required in order to demonstrate the efficacy of the intervention.
